# Inflammatory and Oxidative Biomarkers in Ischemic Stroke

**DOI:** 10.7759/cureus.111972

**Published:** 2026-07-02

**Authors:** Trisha C, Naveen Ch, Prabhat Kumar N, Ramachakra Kushal Thota, Muralidhar Chinnapaka

**Affiliations:** 1 Internal Medicine, Chalmeda AnandRao Institute of Medical Sciences, Karimnagar, IND; 2 General Surgery, Dr. Pinnamaneni Siddhartha Institute of Medical Sciences, Vijayawada, IND; 3 Internal Medicine, Nimra Institute of Medical Sciences, Vijayawada, IND; 4 Internal Medicine, Gandhi Medical College, Secunderabad, IND; 5 Pharmacology, Government Medical College and Hospital Maheshwaram, Hyderabad, IND

**Keywords:** antioxidant enzymes, c-reactive protein, inflammation, interleukin-6, ischemic stroke, malondialdehyde, oxidative stress

## Abstract

Background: Ischemic stroke is a major neurological emergency in which cerebral vessel occlusion triggers a cascade of inflammatory responses, endothelial dysfunction, and oxidative injury. These biochemical changes may influence the extent of neuronal damage and clinical severity. Hence, the present study was undertaken to evaluate selected inflammatory and oxidative stress biomarkers in patients with acute ischemic stroke and to compare their levels with those of age- and sex-matched healthy controls.

Materials and methods: This hospital-based case-control study included 100 subjects, comprising 50 clinically and radiologically confirmed ischemic stroke patients and 50 healthy controls. Blood samples were collected within 24 hours of admission in stroke patients. Serum C-reactive protein (CRP), interleukin-6 (IL-6), tumour necrosis factor-alpha (TNF-α), malondialdehyde (MDA), nitric oxide (NO), superoxide dismutase (SOD), catalase, and reduced glutathione (GSH) were analysed using standard laboratory methods. Stroke severity was assessed using the National Institutes of Health Stroke Scale (NIHSS). Data were analysed using appropriate statistical tests, and p < 0.05 was considered significant.

Results: The mean age of stroke patients was 61.8 ± 10.4 years, while controls had a mean age of 59.6 ± 9.8 years. Serum CRP was significantly higher in stroke patients than in controls (18.6 ± 6.9 mg/L vs. 4.2 ± 1.8 mg/L, p < 0.001). IL-6 (42.5 ± 13.7 pg/mL vs. 12.8 ± 5.4 pg/mL, p < 0.001), TNF-α (31.4 ± 9.6 pg/mL vs. 10.7 ± 4.1 pg/mL, p < 0.001), MDA (6.8 ± 1.9 nmol/mL vs. 2.9 ± 0.8 nmol/mL, p < 0.001), and NO (58.2 ± 14.5 µmol/L vs. 32.6 ± 9.3 µmol/L, p < 0.001) were elevated in cases. Antioxidant markers were reduced, with lower SOD (72.4 ± 18.6 U/mL vs. 118.5 ± 24.3 U/mL), catalase (34.8 ± 9.2 U/mL vs. 56.7 ± 11.4 U/mL), and GSH (5.1 ± 1.4 µmol/L vs. 8.6 ± 2.1 µmol/L) in stroke patients. CRP, IL-6, and MDA showed a positive correlation with NIHSS score. Among the 50 ischemic stroke patients, 14 (28.0%) had mild stroke, 24 (48.0%) had moderate stroke, and 12 (24.0%) had severe stroke based on NIHSS grading.

Conclusion: Ischemic stroke patients showed marked inflammatory activation and oxidative imbalance, reflected by increased CRP, IL-6, TNF-α, MDA, and NO, along with reduced antioxidant defence. These biomarkers may support assessment of stroke severity and vascular injury.

## Introduction

Ischemic stroke remains one of the major causes of death and long-term disability worldwide, with a rising burden in low- and middle-income countries where access to early diagnosis, acute intervention, and structured rehabilitation is often limited [1,2]. It occurs when cerebral blood flow is interrupted by thrombotic or embolic occlusion, leading to oxygen and glucose deprivation in the affected brain tissue. The ischemic core undergoes rapid and often irreversible injury, while the surrounding penumbral area remains potentially salvageable during the early therapeutic window [3,4]. Although neuroimaging is essential for confirming diagnosis and guiding treatment, biochemical markers may provide additional information regarding vascular injury, inflammatory activation, oxidative stress, and disease severity [5].

The pathophysiology of ischemic stroke is not limited to mechanical vessel occlusion. Soon after the ischemic event, a complex cascade involving excitotoxicity, mitochondrial dysfunction, calcium overload, free radical generation, blood-brain barrier disruption, and immune activation is initiated [6,7]. Reperfusion, although essential for restoring blood flow, can further increase oxidative injury through the sudden generation of reactive oxygen species. This oxidative burden damages membrane lipids, proteins, and nucleic acids, thereby worsening neuronal injury and endothelial dysfunction [8,9]. Malondialdehyde (MDA), a product of lipid peroxidation, is commonly used as an indicator of oxidative membrane damage, while antioxidant parameters such as superoxide dismutase (SOD), catalase, and reduced glutathione (GSH) reflect endogenous defence against free radical injury [10,11].

Inflammation is another central mechanism in acute ischemic stroke. Cerebral ischemia activates resident microglia and astrocytes and promotes recruitment of circulating leukocytes into the injured tissue. This response results in increased release of inflammatory mediators such as C-reactive protein (CRP), interleukin-6 (IL-6), tumour necrosis factor-alpha (TNF-α), interleukin-1β, and adhesion molecules [12,13]. These mediators may aggravate endothelial injury, increase vascular permeability, promote thrombosis, and contribute to infarct expansion. At the same time, inflammation also has a role in tissue repair and post-stroke remodelling, making its interpretation biologically complex [14]. Therefore, measurement of inflammatory biomarkers in the acute phase may help in understanding the intensity of the systemic and neurovascular response after stroke.

Among inflammatory markers, CRP is widely available and reflects acute-phase systemic inflammation. Elevated CRP has been associated with vascular risk, larger infarct burden, early neurological worsening, and poor functional recovery in several studies [12,15]. IL-6 is considered an important upstream cytokine that stimulates hepatic production of acute-phase reactants and participates in endothelial activation. TNF-α contributes to leukocyte adhesion, blood-brain barrier disruption, neuronal apoptosis, and amplification of the inflammatory cascade [13,16]. These markers, when assessed together, may provide a broader view of the inflammatory profile than any single biomarker alone.

Oxidative stress and inflammation are closely interlinked in ischemic stroke. Reactive oxygen species can activate inflammatory signalling pathways, while inflammatory cells further enhance oxidative injury through respiratory burst and cytokine-mediated endothelial damage [8,17]. This reciprocal relationship suggests that a combined assessment of inflammatory and oxidative biomarkers may be more informative than evaluating either pathway separately. Such a biomarker panel may help identify patients with greater biochemical injury, support clinical severity assessment, and generate evidence for future risk stratification models [18].

Despite advances in acute stroke care, biomarker-based assessment has not yet become part of routine clinical decision-making. This is partly because many studies vary in sample size, timing of blood collection, biomarker selection, assay methods, and clinical outcome measures [11,18]. More hospital-based studies are needed, particularly from Indian settings, to understand the pattern of inflammatory and oxidative stress markers in patients with acute ischemic stroke. The present study was therefore undertaken to evaluate selected inflammatory and oxidative stress markers in patients with acute ischemic stroke and to compare them with age- and sex-matched healthy controls. The study also explored their association with NIHSS-based stroke severity, as these markers may provide supportive information for assessing inflammatory burden, oxidative imbalance, and possible prognostic risk, rather than serving as direct therapeutic indicators at present.

## Materials and methods

Study design

This was a hospital-based case-control study conducted to evaluate inflammatory and oxidative stress biomarkers in patients diagnosed with acute ischemic stroke. The biomarker profile of ischemic stroke patients was compared with that of apparently healthy age- and sex-matched controls. The study was designed to assess whether acute ischemic stroke was associated with increased inflammatory activity and oxidative imbalance.

Place and duration of study

The study was carried out at Chalmeda AnandRao Institute of Medical Sciences. The study period extended from July 2024 to December 2025. Patients presenting to the hospital with clinical features suggestive of acute ischemic stroke were screened during this period and enrolled after confirmation of diagnosis and fulfilment of eligibility criteria.

Study population

The study population included patients admitted with acute ischemic stroke and healthy controls selected from hospital staff, patient attendants, or individuals attending routine health check-ups. The case group consisted of patients with clinically suspected stroke confirmed by neuroimaging. The control group included age- and sex-matched individuals without a history of stroke, transient ischemic attack, recent infection, chronic inflammatory disease, or major systemic illness.

Sample size

A total of 100 participants were included in the study, comprising 50 patients with acute ischemic stroke and 50 healthy controls. The sample size was considered adequate for comparing mean biomarker levels between the two groups and for exploring the relationship between biomarker levels and stroke severity.

Inclusion criteria

Patients aged 18 years and above with a first episode of acute ischemic stroke confirmed by computed tomography or magnetic resonance imaging were included in the study. Only patients presenting within 24 hours of symptom onset were considered so that early inflammatory and oxidative changes could be assessed. Healthy age- and sex-matched individuals were included as controls after clinical evaluation.

Exclusion criteria

Patients with haemorrhagic stroke, recurrent stroke, transient ischemic attack, active infection, autoimmune disease, chronic inflammatory disorder, malignancy, severe renal disease, severe liver disease, recent surgery, trauma, or myocardial infarction were excluded. Patients receiving antioxidant supplementation, corticosteroids, immunosuppressive drugs, or anti-inflammatory medication before admission were also excluded, as these factors could alter biomarker levels.

Clinical evaluation

After enrolment, a detailed clinical history was obtained from each participant or from the patient's attendant when the patient was unable to provide information. Details regarding age, sex, hypertension, diabetes mellitus, smoking, alcohol intake, dyslipidaemia, previous cardiovascular disease, medication history, and time of onset of symptoms were recorded. General physical examination and systemic examination were performed in all participants. Neurological examination was carried out in stroke patients. Stroke severity was assessed using the National Institutes of Health Stroke Scale (NIHSS), a standardised neurological deficit scale developed for systematic clinical assessment of patients with acute stroke and widely used to quantify neurological impairment in ischemic stroke. Based on NIHSS grading, stroke severity was categorised as mild, moderate, or severe [3,19].

Diagnosis of ischemic stroke

The diagnosis of ischemic stroke was made based on the sudden onset of focal neurological deficit lasting more than 24 hours, supported by neuroimaging evidence. Computed tomography or magnetic resonance imaging of the brain was used to differentiate ischemic stroke from haemorrhagic stroke and other stroke mimics. Only patients with imaging-confirmed ischemic stroke were included in the final analysis.

Blood sample collection

Venous blood samples were collected from stroke patients within 24 hours of hospital admission, preferably before initiation of major therapeutic interventions wherever feasible. In controls, blood samples were collected after clinical screening. Under aseptic precautions, blood was drawn into appropriate plain and anticoagulant-containing vacutainers. Serum or plasma was separated by centrifugation and used for biochemical analysis. Samples meant for delayed analysis were stored under suitable temperature conditions until testing.

Estimation of inflammatory biomarkers

The inflammatory markers assessed in the study included C-reactive protein, interleukin-6, and tumour necrosis factor-alpha. Serum C-reactive protein was measured using a standard immunoturbidimetric or equivalent validated method, depending on laboratory availability. Interleukin-6 and tumour necrosis factor-alpha were estimated using enzyme-linked immunosorbent assay kits according to the manufacturer's instructions. All procedures were performed with appropriate calibration, internal quality control, and duplicate testing wherever applicable.

Estimation of oxidative stress markers

Oxidative stress was assessed by measuring malondialdehyde and nitric oxide levels. Malondialdehyde, a marker of lipid peroxidation, was estimated using the thiobarbituric acid reactive substances method or an equivalent validated assay. Nitric oxide was assessed indirectly by measuring stable nitrate and nitrite levels using a standard colourimetric method. These markers were selected to reflect oxidative membrane injury and altered endothelial-related nitric oxide metabolism in ischemic stroke.

Estimation of antioxidant parameters

The antioxidant defence status was evaluated by measuring superoxide dismutase, catalase, and reduced glutathione. Superoxide dismutase activity was estimated using a standard spectrophotometric method based on inhibition of the superoxide-mediated reaction. Catalase activity was assessed by measuring the decomposition rate of hydrogen peroxide. Reduced glutathione was estimated using a colourimetric method based on reaction with suitable chromogenic reagents. All biochemical estimations were performed in accordance with standard laboratory protocols.

Data collection and variables

Data were collected using a predesigned proforma. The main study variables included demographic details, vascular risk factors, stroke severity score, inflammatory biomarkers, oxidative stress markers, and antioxidant parameters. The primary comparison was between ischemic stroke patients and healthy controls. A secondary analysis was planned to examine the association between biomarker levels and stroke severity among cases.

Statistical analysis

Data were first entered in Microsoft Excel (Microsoft Corporation, Redmond, Washington) and then analysed using IBM SPSS Statistics for Windows, Version 20 (Released 2019; IBM Corp., Armonk, New York). Continuous variables were summarised as mean ± standard deviation. Categorical variables were expressed as frequency and percentage. The normality of continuous data was assessed before comparison. For variables with an approximately normal distribution, the independent-samples t-test was used to compare cases and controls. For variables that were not normally distributed, the Mann-Whitney U test was applied. Categorical variables were compared using the chi-square test, and Fisher's exact test was used when the expected cell count was small. The relationship between biomarker levels and NIHSS-based stroke severity was assessed using Pearson's correlation coefficient for normally distributed variables and Spearman's rank correlation coefficient for non-parametric data. A p-value of less than 0.05 was considered statistically significant.

Ethical considerations

The study was conducted after obtaining approval from the Institutional Ethics Committee of Chalmeda AnandRao Institute of Medical Sciences. Written informed consent was obtained from all participants or from legally acceptable representatives in cases where patients were unable to provide consent. Confidentiality of participant information was maintained throughout the study, and all procedures were carried out in accordance with ethical principles for biomedical research involving human participants.

## Results

Baseline characteristics of the study participants

The study included 100 participants, with 50 patients diagnosed with acute ischemic stroke and 50 age- and sex-matched healthy controls. The mean age of the ischemic stroke group was 61.8 ± 10.4 years, while the control group had a mean age of 59.6 ± 9.8 years. The difference in age between the two groups was not statistically significant. Males constituted 28/50 (56.0%) of the control group and 31/50 (62.0%) of the ischemic stroke group. Although males were slightly more frequent in both groups, the sex distribution was comparable between the groups, and the difference was not statistically significant (χ² = 0.37, p = 0.542). Hypertension, diabetes mellitus, smoking, and dyslipidaemia were more frequent among ischemic stroke patients than among controls, suggesting a higher vascular risk burden in the case group (Table 1). 

**Table 1:** Baseline characteristics of ischemic stroke patients and controls Values are expressed as mean ± SD or number with percentage. Independent sample t-test was used for age comparison. Chi-square test was used for categorical variables. p < 0.05 was considered statistically significant.

Variable	Controls, n = 50	Ischemic stroke, n = 50	p-value/χ²-value
Age, years, mean ± SD	59.6 ± 9.8	61.8 ± 10.4	p = 0.279
Male sex, n (%)	28 (56.0%)	31 (62.0%)	χ² = 0.37, p = 0.542
Female sex, n (%)	22 (44.0%)	19 (38.0%)	χ² = 0.37, p = 0.542
Hypertension, n (%)	12 (24.0%)	31 (62.0%)	χ² = 14.75, p < 0.001
Diabetes mellitus, n (%)	9 (18.0%)	22 (44.0%)	χ² = 7.89, p = 0.005
Smoking, n (%)	8 (16.0%)	21 (42.0%)	χ² = 8.21, p = 0.004
Dyslipidaemia, n (%)	10 (20.0%)	24 (48.0%)	χ² = 8.73, p = 0.003

Inflammatory biomarker profile

Inflammatory markers were markedly increased in patients with acute ischemic stroke compared with healthy controls. Mean C-reactive protein (CRP) level was 18.6 ± 6.9 mg/L in stroke patients and 4.2 ± 1.8 mg/L in controls. Similarly, IL-6 and TNF-α levels were significantly higher among cases (Table 2).

**Table 2:** Comparison of inflammatory biomarkers between ischemic stroke patients and controls Values are expressed as mean ± SD. A comparison was performed using an independent-samples t-test. CRP: C-reactive protein; IL-6: interleukin-6; TNF-α: tumour necrosis factor-alpha.

Biomarker	Controls, n = 50	Ischemic stroke, n = 50	p-value
CRP, mg/L	4.2 ± 1.8	18.6 ± 6.9	<0.001
IL-6, pg/mL	12.8 ± 5.4	42.5 ± 13.7	<0.001
TNF-α, pg/mL	10.7 ± 4.1	31.4 ± 9.6	<0.001

**Figure 1:**
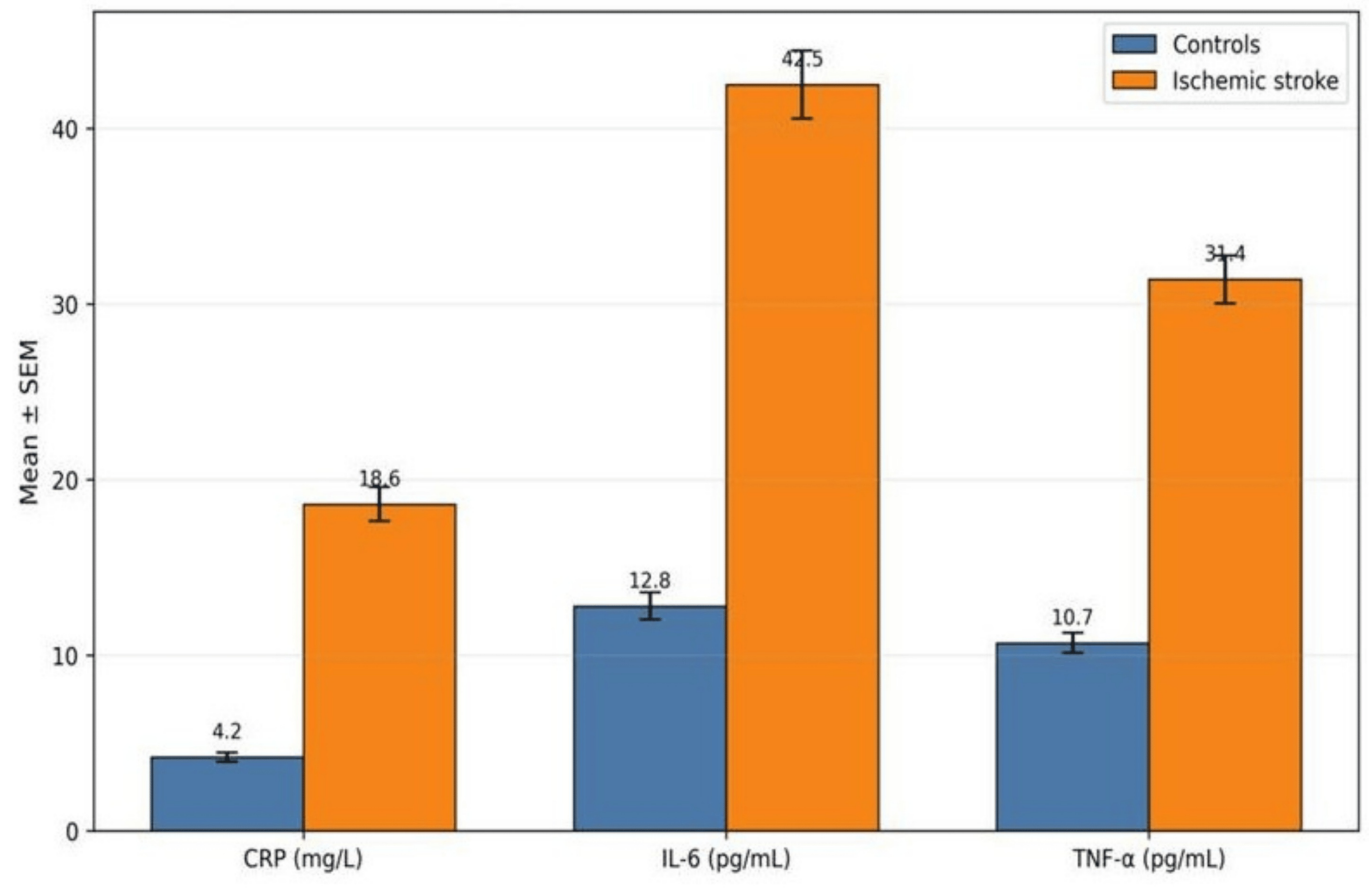
A comparison of inflammatory biomarkers between controls and ischemic stroke patients. Stroke patients showed significantly higher CRP, IL-6, and TNF-α levels, indicating increased inflammatory activity during the acute phase of ischemic stroke CRP: C-reactive protein; IL-6: interleukin-6; TNF-α: tumour necrosis factor-alpha.

These findings indicate strong systemic inflammatory activation during the acute phase of ischemic stroke (Figure 1).

Oxidative stress markers

Oxidative stress markers were significantly elevated in the ischemic stroke group. The mean MDA level was 6.8 ± 1.9 nmol/mL in stroke patients, compared with 2.9 ± 0.8 nmol/mL in controls. Nitric oxide levels were also higher in cases than in controls (Table 3).

**Table 3:** Comparison of oxidative stress markers between ischemic stroke patients and controls Values are expressed as mean ± SD. Comparison was performed using independent sample t-test. MDA: malondialdehyde.

Biomarker	Controls, n = 50	Ischemic stroke, n = 50	p value
MDA, nmol/mL	2.9 ± 0.8	6.8 ± 1.9	<0.001
Nitric oxide, µmol/L	32.6 ± 9.3	58.2 ± 14.5	<0.001

**Figure 2:**
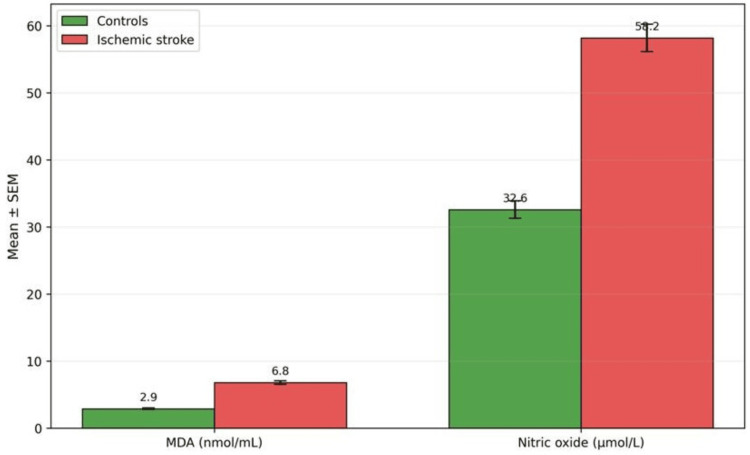
Oxidative stress markers in controls and ischemic stroke patients. MDA and nitric oxide were higher in the stroke group, supporting the presence of increased oxidative burden after cerebral ischemia MDA: malondialdehyde.

This pattern suggests increased lipid peroxidation and altered nitric oxide metabolism in acute ischemic stroke (Figure 2).

Antioxidant status

Antioxidant parameters were significantly reduced in patients with ischemic stroke compared with controls. Mean superoxide dismutase (SOD) activity was 72.4 ± 18.6 U/mL in cases and 118.5 ± 24.3 U/mL in controls. Catalase and reduced glutathione levels were also lower among stroke patients (Table 4).

**Table 4:** Comparison of antioxidant parameters between ischemic stroke patients and controls Values are expressed as mean ± SD. Comparison was performed using independent sample t-test. SOD: superoxide dismutase.

Antioxidant parameter	Controls, n = 50	Ischemic stroke, n = 50	p-value
SOD, U/mL	118.5 ± 24.3	72.4 ± 18.6	<0.001
Catalase, U/mL	56.7 ± 11.4	34.8 ± 9.2	<0.001
Reduced glutathione, µmol/L	8.6 ± 2.1	5.1 ± 1.4	<0.001

**Figure 3:**
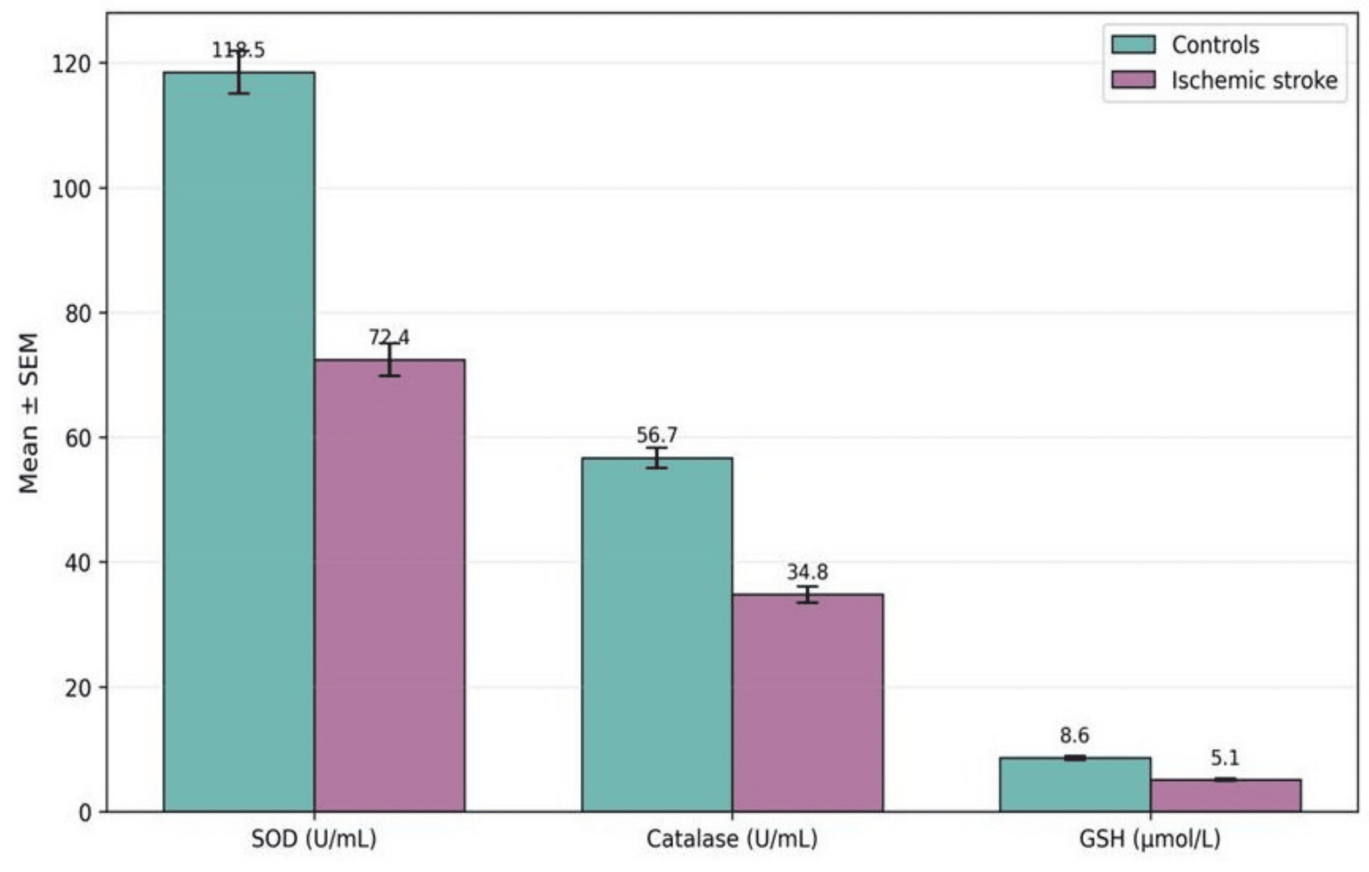
Antioxidant parameters in controls and ischemic stroke patients. SOD, catalase, and reduced glutathione were significantly lower in the stroke group, reflecting impaired antioxidant protection SOD: superoxide dismutase.

These findings suggest depletion of antioxidant defence mechanisms in response to acute oxidative injury (Figure 3).

Stroke severity distribution

Among the 50 ischemic stroke patients, 14 (28%) had a mild stroke, 24 (48%) had a moderate stroke, and 12 (24%) had a severe stroke based on the National Institutes of Health Stroke Scale (NIHSS) grading [19]. Moderate stroke was the most common clinical severity category in the study population. Patients with severe stroke showed higher inflammatory and oxidative biomarker levels than those with mild stroke (Table 5). 

**Table 5:** Distribution of ischemic stroke patients according to NIHSS severity NIHSS: National Institutes of Health Stroke Scale.

Stroke severity	NIHSS score range	Number of patients	Percentage
Mild stroke	1-4	14	28.0%
Moderate stroke	5-15	24	48.0%
Severe stroke	>15	12	24.0%

Biomarker pattern across stroke severity

A stepwise rise in inflammatory and oxidative stress markers was observed with increasing stroke severity. Patients with severe stroke had the highest CRP, IL-6, TNF-α, and MDA levels. In contrast, antioxidant markers showed a gradual decline from mild to severe stroke. This trend suggests that greater neurological deficit was associated with stronger inflammatory activation and oxidative imbalance (Table 6). 

**Table 6:** Biomarker levels according to stroke severity among ischemic stroke patients Values are expressed as mean ± SD. One-way ANOVA was used to compare biomarker levels across stroke severity categories. p < 0.05 was considered statistically significant. CRP: C-reactive protein, IL-6: interleukin-6, TNF-α: tumour necrosis factor-alpha, MDA: malondialdehyde, SOD: superoxide dismutase, n: number.

Biomarker	Mild stroke, n = 14	Moderate stroke, n = 24	Severe stroke, n = 12	p-value
CRP, mg/L	12.8 ± 4.1	18.9 ± 5.7	25.1 ± 6.8	<0.001
IL-6, pg/mL	31.6 ± 9.2	42.8 ± 11.4	55.3 ± 12.6	<0.001
TNF-α, pg/mL	23.4 ± 6.8	31.5 ± 8.2	40.6 ± 9.1	<0.001
MDA, nmol/mL	5.1 ± 1.2	6.9 ± 1.5	8.4 ± 1.8	<0.001
SOD, U/mL	86.5 ± 16.2	71.8 ± 15.4	56.9 ± 13.7	<0.001
Catalase, U/mL	42.6 ± 8.3	34.1 ± 7.8	27.3 ± 6.9	<0.001
Reduced glutathione, µmol/L	6.2 ± 1.1	5.0 ± 1.2	4.1 ± 0.9	<0.001

Correlation between biomarkers and NIHSS score

Correlation analysis showed that CRP, IL-6, TNF-α, MDA, and nitric oxide had a positive correlation with NIHSS score. Among these, CRP, IL-6, and MDA showed stronger associations with stroke severity. Antioxidant markers such as SOD, catalase, and reduced glutathione showed a negative correlation with NIHSS score, suggesting that antioxidant depletion was greater in patients with more severe neurological involvement (Table 7). 

**Table 7:** Correlation of biomarkers with NIHSS score among ischemic stroke patients Pearson’s correlation test was used. NIHSS: National Institutes of Health Stroke Scale; CRP: C-reactive protein; IL-6: interleukin-6; TNF-α: tumour necrosis factor-alpha; MDA: malondialdehyde; SOD: superoxide dismutase.

Biomarker	Correlation coefficient, r	p-value
CRP	0.62	<0.001
IL-6	0.58	<0.001
TNF-α	0.49	<0.001
MDA	0.64	<0.001
Nitric oxide	0.41	0.003
SOD	-0.55	<0.001
Catalase	-0.47	0.001
Reduced glutathione	-0.52	<0.001

## Discussion

The present study demonstrated that acute ischemic stroke was associated with a marked inflammatory and oxidative response. Patients showed significantly higher serum CRP, IL-6, TNF-α, MDA, and nitric oxide levels, along with reduced SOD, catalase, and reduced glutathione when compared with healthy controls. This pattern suggests that ischemic stroke is not merely a vascular occlusive event, but a complex biochemical condition involving inflammation, endothelial dysfunction, lipid peroxidation, and depletion of antioxidant defence.

CRP was markedly elevated among stroke patients, indicating activation of systemic inflammation during the acute phase. As an acute-phase protein, CRP reflects vascular inflammation, endothelial injury, and tissue damage following cerebral ischemia. Similar findings have been reported earlier, where raised inflammatory markers were linked with stroke severity, infarct progression, and clinical outcome [12,15]. The increased CRP observed in this study therefore supports its role as a simple and useful indicator of inflammatory activity in acute ischemic stroke.

The significant rise in IL-6 and TNF-α further confirms cytokine-mediated inflammatory activation. IL-6 promotes the hepatic acute-phase response and endothelial activation, while TNF-α contributes to leukocyte recruitment, blood-brain barrier disruption, and propagation of ischemic injury [13,16]. Following cerebral ischemia, activated microglia, astrocytes, and infiltrating immune cells release these cytokines into the ischemic tissue and circulation [6,7]. Thus, the higher IL-6 and TNF-α levels in the present study may reflect ongoing neuroinflammation and secondary neuronal damage.

Oxidative stress was another important finding. MDA levels were significantly increased in stroke patients, suggesting enhanced lipid peroxidation. During ischemia, impaired oxygen and glucose delivery leads to mitochondrial dysfunction, excitotoxicity, and excessive generation of reactive oxygen species [8,9]. These radicals damage membrane lipids, particularly polyunsaturated fatty acids, resulting in MDA formation. Previous studies have also shown increased oxidative biomarkers after ischemic stroke and their association with disease severity and prognosis [10,11]. Therefore, elevated MDA in the present study indicates active oxidative membrane injury during the acute stage.

Nitric oxide was also higher in the stroke group. Although nitric oxide has a physiological role in maintaining vascular tone and cerebral perfusion, excessive production during ischemia and reperfusion may become harmful. It can react with superoxide radicals to form peroxynitrite, which damages proteins, lipids, and DNA [8,17]. The raised nitric oxide levels observed in this study may therefore represent endothelial stress and nitrosative injury, supporting the involvement of both oxidative and nitrosative mechanisms in ischemic neuronal damage.

The reduction in SOD, catalase, and reduced glutathione indicates weakening of the endogenous antioxidant system. SOD converts superoxide radicals into hydrogen peroxide, catalase further breaks down hydrogen peroxide, and reduced glutathione helps maintain intracellular redox balance. Lower levels of these antioxidants suggest that protective mechanisms were either consumed or overwhelmed during acute ischemic injury [9,10]. Similar reductions in antioxidant capacity have been described in ischemic stroke, strengthening the view that oxidative imbalance is central to stroke pathophysiology [11].

A notable observation was the association between biomarker levels and NIHSS score. CRP, IL-6, TNF-α, MDA, and nitric oxide showed a positive relationship with stroke severity, whereas SOD, catalase, and reduced glutathione showed an inverse relationship. This indicates that patients with more severe neurological impairment had greater inflammatory and oxidative burden, together with poorer antioxidant protection. Earlier reports also suggest that combined evaluation of inflammatory and oxidative markers may provide additional information on stroke severity and prognosis [5,18]. Although these biomarkers cannot replace clinical assessment or neuroimaging, they may support a better understanding of the biological severity of stroke.

Inflammation and oxidative stress are closely connected in ischemic stroke. Reactive oxygen species can activate inflammatory pathways, while inflammatory cells can intensify oxidative injury through cytokine release and respiratory burst activity [8,17]. This creates a damaging cycle that may aggravate endothelial dysfunction, blood-brain barrier injury, cerebral oedema, neuronal apoptosis, and infarct expansion [6,14]. The simultaneous increase in inflammatory and oxidative markers in the present study supports this interlinked mechanism.

These findings may have clinical relevance. Measurement of selected biomarkers may help identify patients with higher biochemical stress after ischemic stroke. CRP and cytokines reflect inflammatory activation, while MDA, nitric oxide, and antioxidant markers provide insight into oxidative injury and redox imbalance. When combined with NIHSS score and imaging findings, these markers may contribute to future risk stratification models [5,15]. However, their routine clinical use requires validation in larger prospective studies.

The study has a few limitations that should be considered while interpreting the findings. This was a hospital-based case-control study with a relatively modest sample size, which may limit the wider applicability of the results. Biomarkers were measured only once during the acute phase, so changes over time during recovery or clinical worsening could not be assessed. The study also did not examine the correlation with infarct volume, stroke subtype, or long-term functional outcome in detail. In addition, common vascular risk factors such as diabetes mellitus, hypertension, smoking, and dyslipidaemia may independently affect inflammatory and oxidative stress markers and could have influenced the observed biomarker levels. Despite these limitations, the study provides useful supportive evidence that acute ischemic stroke is associated with inflammatory activation, oxidative stress, and depletion of antioxidant defence, with these changes becoming more evident as stroke severity increases. Larger prospective studies with serial biomarker estimation, adjustment for comorbidities, and follow-up outcome analysis are needed to confirm the prognostic value of these markers in ischemic stroke.

Overall, the study reinforces the role of inflammation and oxidative stress in acute ischemic stroke. Increased CRP, IL-6, TNF-α, MDA, and nitric oxide, along with reduced SOD, catalase, and reduced glutathione, indicate a clear shift toward inflammatory and oxidative injury. Their association with the NIHSS score suggests that biomarker imbalance may reflect clinical severity. Further studies with larger cohorts, serial biomarker estimation, and follow-up outcome assessment are needed to clarify their prognostic value in ischemic stroke.

## Conclusions

The present study found that acute ischemic stroke was associated with increased inflammatory activity and oxidative stress. Patients with ischemic stroke showed higher serum levels of CRP, IL-6, TNF-α, MDA, and nitric oxide, along with lower antioxidant markers such as SOD, catalase, and reduced glutathione compared with healthy controls. These changes were more marked in patients with greater NIHSS-based stroke severity, suggesting that biomarker imbalance may reflect the biological burden of ischemic injury. Although these markers cannot replace neurological examination or neuroimaging, they may provide useful supportive information for assessing disease severity, vascular injury, and possible prognostic risk. The findings also support the role of inflammation, oxidative damage, and antioxidant depletion in the pathophysiology of ischemic stroke. Further large-scale prospective studies with serial biomarker estimation, adjustment for comorbidities, and long-term outcome assessment are needed to confirm their clinical and prognostic utility in routine stroke care.
